# Diabetes distress in Indonesian patients with type 2 diabetes: a comparison between primary and tertiary care

**DOI:** 10.1186/s12913-019-4515-1

**Published:** 2019-10-30

**Authors:** Bustanul Arifin, Antoinette D. I. van Asselt, Didik Setiawan, Jarir Atthobari, Maarten J. Postma, Qi Cao

**Affiliations:** 1 Unit of Pharmacotherapy, Epidemiology & Economics (PTE2), Department of Pharmacy, University of Groningen, Groningen, the Netherlands; 2 Disease Prevention and Control Division, Banggai Laut Regency Health, Population Control and Family Planning Service, Central Sulawesi, Indonesia (Bidang Pencegahan dan Pengendalian Penyakit, Dinas Kesehatan, Pengendalian Penduduk & Keluarga Berencana, Pemerintah Daerah Kabupaten Banggai Laut, Jl. Jogugu Zakaria No. 1, Banggai, Sulawesi Tengah, Indonesia; 3 Institute of Science in Healthy Ageing & healthcaRE (SHARE), University Medical Center Groningen (UMCG), University of Groningen, Groningen, the Netherlands; 4 Department of Health Sciences, University of Groningen, University Medical Center Groningen, University of Groningen, Hanzeplein 1, Groningen, 9700 RB the Netherlands; 5 Department of Epidemiology, University of Groningen, University Medical Center Groningen, Groningen, the Netherlands; 6 Faculty of Pharmacy, Universitas Muhammadiyah Purwokerto, Purwokerto, Indonesia; 7 Department of Pharmacology and Therapy, Faculty of Medicine, Public Health and Nursing, Universitas Gadjah Mada, Yogyakarta, Indonesia; 8 Clinical Epidemiology and Biostatsitic Unit, Faculty of Medicine, Public Health and Nursing, Universitas Gadjah Mada, Yogyakarta, Indonesia; 9 Department of Economics, Econometrics & Finance, Faculty of Economics & Business, University of Groningen, Groningen, the Netherlands; 10 Department of Pharmacology, Universitas Airlangga, Surabaya, Indonesia

**Keywords:** Diabetes distress, Indonesia, Primary care, Tertiary care

## Abstract

**Background:**

The number of people living with diabetes mellitus (DM) in Indonesia has continued to increase over the last 6 years. Four previous studies in U.S have found that higher DD scores were associated with worse psychological outcomes, lower health-related quality of life (HRQoL) and increased risk of T2DM complications. In this study, we aimed to firstly compare DD scores in Indonesian T2DM outpatients treated in primary care versus those in tertiary care. Subsequently, we investigated whether socio-demographic characteristics and clinical conditions explain potential differences in DD score across healthcare settings.

**Methods:**

A cross-sectional study was conducted on Java island in three primary care (*n* = 108) and four tertiary care (*n* = 524) facilities. The participants completed the Bahasa Indonesia version of the Diabetes Distress Scale questionnaire (DDS17 Bahasa Indonesia). Ordinal regression analysis was conducted with the quartile of the summation of the DD score as the dependent variable to investigate how the association between the level of healthcare facilities and DD altered when adding different variables in the model.

**Results:**

The final adjusted model showed that the level of healthcare facilities was strongly associated with DD (*p* < .001), with participants in primary care having a 3.68 times (95% CI 2.46–5.55) higher likelihood of being more distressed than the participants in tertiary care. This association was detected after including the socio-demographic characteristics and clinical conditions as model confounders.

**Conclusions:**

This is the first study in Indonesia to compare DD scores within different healthcare facilities. We recommend a regular DD assessment, possibly closely aligned with health-literacy partner programs, especially for T2DM patients in primary care settings.

## Background

The number of people living with diabetes mellitus (DM) in Indonesia has continued to increase over the last 6 years. In 2009, the International Diabetes Federation (IDF) estimated that there were around 7.3 million people living with type 2 DM (T2DM) and in 2017 this number increased to 10.3 million, among whom 7.3 million are undiagnosed [1, 2]. The percentage of persons living with T2DM was higher in females than in males (7.7% vs. 5.6%), and new cases were not only found in the above-55-years age group but also in younger age groups (starting at 15 years of age). With regard to the level of education, the highest prevalence was found in community groups who had never attended school (10.4%) [3].

Diabetes distress (DD) is a psychological condition which overlaps with anxiety, stress and depression [4]. In 2005, Polonsky et al. published an assessment of psychological distress in diabetes patients in three cities in the U.S (San Diego, Boston, and Honolulu) [5]. Based on that research, Polonsky et al. have developed the DD instrument labelled DDS (diabetes distress scale), focussing on assessing DD in four domains: physician distress, emotional burden, regimen distress, and interpersonal distress [6]. In total, 28 items are covered in these 4 domains. In 2012, Fisher et al. have conducted further research to evaluate the association of the 28 DDS items with the clinical condition of their participants [7]. Based on the associations, the latest version of DDS could be reduced to only 17 items [5, 7]. DD assessment is highly recommended because it complements other T2DM assessments, enabling a more comprehensive approach both clinically as well as psychologically [6, 8].

Studies comparing DD scores across healthcare settings are currently limited. We found two studies in the Netherlands and Greece [9, 10], however, we have not found any similar study for Indonesia. Both of these studies reported that the DD scores of participants treated in tertiary care were higher than those in primary care. The Dutch study reported that besides the care setting, other factors such as younger age, ethnic minority status, use of insulin, higher HbA1c levels, higher body mass index (BMI) and neuropathy were associated with higher DD scores. Furthermore, the Greek study reported that comorbidities, the use of insulin, and T2DM duration were associated with higher DD scores.

This research is important to be carried out in Indonesia because so far T2DM therapy is focused almost solely on the clinical aspects. It is important to note that recent studies have proven that psychological conditions of T2DM patients also highly influence levels of glycemic control and overall well-being [10]. Furthermore, this research can be used as a scientific base for the government of Indonesia in organizing T2DM programs, especially in supporting the government program to strengthen the primary service in Indonesia. In this study, we aimed to firstly compare DD scores in Indonesian T2DM outpatients treated in primary care versus those in tertiary care. Subsequently, we investigated whether socio-demographic characteristics and clinical conditions explain potential differences in DD scores across healthcare settings.

## Methods

### T2DM management in Indonesia

In Indonesia, the national health insurance is known as ‘BPJS (Badan Penyelenggara Jaminan Sosial)/ Social Security Administrative Agency’. Since January 1st, 2014, T2DM patients’ care in Indonesia has been managed by a referral system, where all T2DM patients will receive initial care in primary care (Puskesmas/ public healthcare center (PHC)) and by a family doctor [11]. Determination of the location of primary care is based on the location where the T2DM patients live, or by request of T2DM patients themselves [11]. At primary care level, the BPJS also has a chronic disease management program labelled Prolanis, which is an integrated and tiered T2DM patient service program aimed at improving the quality of life of T2DM patients in Indonesia [12]. Besides BPJS’s Prolanis, another community-based organization for T2DM patients exists, labelled Persadia (Indonesian Diabetes Association) [13]. Prolanis and Persadia both have the same goal. Therefore T2DM patients can follow the activities of both communities.

### Research setting

#### Primary care

In our study, we defined primary care as the setting where T2DM outpatients are managed by a GP. Generally and additionally, every 6 months they have an opportunity to consult with a resident of internal medicine in a tertiary care facility (see below for exact definition).

#### Tertiary care

In tertiary care, T2DM outpatients are treated in a hospital setting and monitored by a consulting resident of internal medicine. During the monitoring process, the resident of internal medicine plans the therapy according to guidelines, including the prescription of insulin for T2DM outpatients whose blood glucose remains uncontrolled with oral antidiabetic drugs (OADs) therapy. Insulin administration may continue for a certain period of time until the patient shows better clinical outcomes, for instance, his/her blood glucose is brought under control (for example, fasting blood glucose (FBG) ≤126 mg/dL) and afterwards, therapy may be reverted from insulin to OAD. Then, the consulting resident of internal medicine refers the patients back to a primary care facility for continuing OAD therapy. However, there are also some T2DM outpatients whose insulin therapy cannot be replaced by OAD, i.e. those who report OAD side effects or are in continued need of insulin [14]. For this group of patients, therapy continues in tertiary care and they continue to be monitored by a resident of internal medicine. In areas with limited internal medicine facilities, T2DM outpatients with insulin therapy can take routine examinations in a primary care facility and get the insulin at private pharmacies that collaborate with the BPJS.

### Research context

Generally, health care facilities in primary and tertiary care settings in Indonesia are public ones in which all health service procedures are managed by the government of Indonesia. In this research, all participants were covered with health insurance recommended by the government.

### Study design and research sites

A cross-sectional study was conducted between February 2015 and April 2016. The period of this study was determined by the health facility. Each researcher who was going to collaborate with them would then be scheduled so that in one period of data collection there would be no more than one researcher (especially with topics and methods of collecting data that were almost the same). In primary care settings, participants were selected from a family doctor in Wonosari in Yogyakarta, a PHC in Surabaya (East Java) and a T2DM outpatients’ community in Surakarta (Central Java). Data collection was done every Friday and Saturday during a weekly patient education program where T2DM outpatients gather for weekly exercise and education about T2DM. In tertiary care settings, participants were selected from RSUD Kota Yogyakarta Hospital, PKU Muhammadiyah Hospital in Yogyakarta, Moewardi Hospital in Surakarta (Central Java) and BLUD RSUD Sekarwangi Hospital in Sukabumi (West Java). Most of the participants were interviewed in the waiting rooms of the hospitals while they were waiting for a consultation with a consulting resident of internal medicine. All the hospitals in this study were public teaching hospitals. During their handling of T2DM patients, all the consulting residents were under the supervision of an internist. The remaining participants were questioned in the waiting rooms of the hospitals’ pharmacies. The Medical Ethics Committee of Universitas Gadjah Mada (Yogyakarta, Indonesia) approved the study with document number KE/FK/1188/EC on November 12th, 2014 (the approval was amended on March 16th, 2015 due to the increased number of research sites). Furthermore, The Ministry of Home Affairs of Republic of Indonesia issued a research recommendation (number 440.02/ 4480 on 25 November 2014) and sent it to the provinces and regencies where the proposed research was to be carried out. In addition, we also obtained a research permit from the Central BPJS with document number 856/ Bang/ 0914 on September 17, 2014. Based on this Central BPJS permit, we were able to collaborate with BPJS branches in the provinces and regencies. Data collection in all health facilities obtained permission from relevant parties, such as hospital directors, head of PHCs or family doctors.

### Participants

We included outpatients with T2DM that were older than 18 years. The participants of this study were only those we met during data collection and were willing to read and sign an informed consent and were comfortable with filling out the instrument. Some potential participants refused to participate because: (i) they forgot to bring their glasses; (ii) they felt too tired because of the bureaucracy in the hospital, as some of them had been in the hospital for around 7 h (since 5 am for registration). For participants with limited reading ability or physical limitations, informed consent was given orally by the caregiver (spouses or adult children) and they also gave written consent with their caregiver as witnesses. In this study, all caregivers were aged over 18 years.

### Instrument

We used the Diabetes Distress Scale questionnaire in Bahasa Indonesia (DDS17 Bahasa Indonesia) [15] to measure the DD score. This instrument has been through a translation process, revision and validation into Indonesian language (see Additional files 1 and 2) [15]. DDS17 Bahasa Indonesia consists of 17 items which are divided into four domains. First, three items are specified in the interpersonal distress domain concerning items on support from family members and colleagues of T2DM outpatients. Second, five items specify the emotional burden domain with regard to the concerns and fears of T2DM outpatients concerning complications. Third, four items in the physician distress domain describe opinions of T2DM outpatients concerning the knowledge and attitude of and the care provided by the treating physician. The last five items specified in the regimen distress domain measure difficulties of T2DM outpatients concerning the management of T2DM therapies (including motivation) and issues in self-confidence or stress, for example, caused by routine blood sugar checks. Each item consists of a scale ranging from 1 (not a problem) to 6 (a very serious problem) [5]. The resulting sum score of the 17 items would then range from 17, ‘not a problem’ to 102, ‘very serious problem’ [5, 7]. According to the revised DDS scores developed by Fisher et al. (2012) [7], an overall mean score of less than 2.0 indicates little or no DD, a score between 2.0–2.9 indicates moderate DD and a score of 3.0 or higher indicates high DD.

### Data collection procedure and data source

We collaborated with treating GPs and consulting residents of internal medicine to collect our data. The GPs and residents assisted us by providing participants with information about the research objectives, ethics and the importance of participating. This information helped participants to be more focused and comfortable and strengthened the feeling that the research was supported by the hospital staff. Participants were assisted by the researcher or the research assistant while filling out the questionnaire. We accompanied participants while the instrument was filled out and gave them the opportunity to ask questions. If necessary, information or explanation could be repeated to individual participants, so that all participants have the same understanding of one item. All the research assistants involved in this research got training beforehand from the main researchers. In order to minimize bias, one of the main researchers was always present with the research assistants during the data collection. Furthermore, the main researchers always discussed the data collection procedure before and after the data collection. During the data collection process, the same procedure was followed in the primary care and tertiary care settings.

Socio-demographic characteristics such as age, sex, educational background, and occupation were collected using the participants’ identity cards and from self-reporting. In this study, the age of participants was classified into two categories: younger/productive age (18–56 years) or retirement age (> 56 years). Furthermore, those aged 18–56 years who reported not currently having a job were defined as unemployed. Those who stated their main job is to take care of the household were classified as housewives, even when they were beyond the retirement age. Clinical condition such as the type of therapy, complications and other serious diseases were obtained from the treating GPs and residents of internal medicine. Postprandial blood glucose, FBG and T2DM duration were collected from self-reporting. In this study, participants were defined as having other serious diseases if they suffered from other major diseases besides diabetes, such as cancer or tuberculosis. Also, participants experiencing exclusive T2DM complications, such as hypertension and cardiovascular diseases, were identified as a separate group.

### Statistical analysis

Descriptive statistical analysis was performed to investigate whether socio-demographic characteristics, variables refelcting clinical conditions, and the DD score differed between participants in primary and tertiary care. T-test was performed to compare the normally distributed variables with the mean and standard deviation reported, meanwhile Kruskal-Wallis rank sum tests were performed to compare the variables with skewed distribution (i.e., non-normal) including DD scores with the median and inter-quartile range (IQR) reported. For the categorical variables, the comparison were conducted based on Chi-square test. Ordinal regression analysis was conducted with the quartile of the summation of the DD scores categorized based on its distribution as the dependent variable to investigate how the association between the level of healthcare facilities and the DD score altered when adding different confounding variables into the model. This model was chosen because the distribution of the summation of DD scores is highly skewed and as such a linear regression cannot be performed with a non-normal response variable. A generalized linear model, with ordinal regression as a specific type, was then conducted when the outcome variable was not normally distributed (i.e., skewed). We assessed the existence of multicollinearity in our model by the correlation matrix of all independent variables (r > 0.80 indicates multicollinearity) and the variance inflation factor (a value > 10 indicates multicollinearity). The association between the level of healthcare facilities and DD score was firstly investigated in an unadjusted model (model 1). Subsequently, we investigated how such association altered when adjusting for sex, age, educational level, occupation, and the presence or absence of a caregiver (model 2). We then investigated the alteration by further adding the clinical variables (T2DM duration, FBG) into model 2 (model 3). Finally, complications and other serious diseases were added as variables into model 4 and model 5 (replacing the variables of clinical conditions in model 4 and a full model in model 5). The differences in odds ratios between the models (ΔOR) were subsequently calculated. In addition, we investigated in each multivariate model (model 2 to 5) how the socio-demographic characteristics and clinical variables independently related to the DD score except for the effect of the level of care. We did not include the type of therapy and postprandial glucose into any of our ordinal regression models because adding these variables would cause the resulting models deviating from the proportional odds assumption [16]. Missing values on T2DM duration and FBG were dealt with using multiple imputations [17]. For both the descriptive analysis and the regression model, the complications and other serious diseases were described as dichotomous data (i.e., yes/no one complication, yes/no two or more complications, yes/no other serious diseases). Considering the high percentage of missing measurements, we obtained 50 imputed datasets for each measurement. The completed measures were then computed by taking the average values generated from each imputed dataset. Statistical analyses were performed using R (R Foundation for Statistical Computing, software version 3.4.0, Vienna, Austria). The factors were considered statistically significant coefficients in the regression analyses if the two-tailed *p*-value was <.05.

## Results

### Participants’ characteristics

In total, 632 participants were included in the study, of whom 108 (17%) were from a primary care setting and 524 (83%) were from a secondary care setting (Table 1). Participants in the primary care setting were older and were relatively more frequently housewives. In addition, they had a longer T2DM duration and a lower percentage of suffering from two or more complications and other serious diseases. The total DD score in the primary care setting was shown to be significantly higher than the score in the secondary care setting. This was also the case for the score in each domain.

**Table 1:** Socio-demographic characteristics, clinical condition and diabetes distress scores of the participants in primary care compared to those treated in tertiary care

Variables	Primary care (*n* = 108)	Tertiary care (*n* = 524)	Overall (*n* = 632)	*P*-value
Socio-demographic characteristics				
Male sex	32%	44%	43%	0.235
Age [years]*	62 ± 9	60 ± 10	60 ± 10	0.010
University degree	12%	26%	26%	0.224
Occupation (I/II/III)^a,^***	10%/40%/50%	31%/34%/35%	29%/35%/36%	< 0.001
Accompanied by caregiver	53%	62%	61%	0.132
Clinical variables				
Diabetes duration [years]*	5 (1–14); N = 31	4 (1–10); *N* = 312	5 (1–10); *N* = 343	0.028
Type of therapy (I/II/III/IV)^b^,***	11%/67%/14%/8%	2%/57%/24%/17%	5%/59%/22%/14%	< 0.001
Fasting blood glucose (FBG) [mg/dL]	130 (112–134); *N* = 9	140 (115–179); *N* = 249	140 (115–180); *N* = 258	0.440
Postprandial glucose [mg/dL]	167 (160–184); N = 9	192 (151–236); *N* = 234	190 (153–236); *N* = 243	0.603
Complications				
No complication	47%	33%	32%	0.576
With one complication	33%	37%	36%	0.116
With two or more complications*	17%	23%	26%	0.011
With other serious diseases*	3%	7%	6%	0.011
Diabetes distress				
Total score***	28 (21–41)	21 (18–30)	23 (18–35)	< 0.001
Emotional burden***	8 (6–11)	6 (5–9)	7 (5–10)	< 0.001
Physician distress***	7 (5–10)	5 (4–7)	5 (4–8)	< 0.001
Regimen distress***	9 (6–13)	6 (5–9)	7 (5–11)	< 0.001
Interpersonal distress***	4 (3–6)	3 (3–5)	3 (3–6)	< 0.001

Continuous variables are presented as mean ± standard deviation or median (interquartile range), and categorical variables are presented as percentages

^a^Occupation I, II, III respectively stand for active employee, unemployed, and housewife

^b^Type of therapy I, II, III, IV respectively stand for Diet or no drugs, OAD, Insulin, Insulin+OAD

***P < 0.001;**P < 0.01;*P < 0.05

### Factors explaining differences in DD scores between primary and tertiary care

Table 2 depicts the results of the ordinal regression models. The multicollinearity statistics indicated no significant multicollinearity between the independent variables. The unadjusted model (model 1) showed that the level of health facilities was strongly associated with DD (*p* < .001), with participants in primary care having a 2.91 times (95% CI 1.98–4.29) higher likelihood of being more distressed than the participants in tertiary care. Model 2 showed that after adjusting for socio-demographic characteristic variables, the association was strongly intensified (ΔOR = 0.48). In Model 3, adding the clinical condition variables further intensified the association between the level of health facilities and DD (ΔOR = 0.09). In Model 4, replacing clinical variables with complications showed a moderately intensified association compared to model 2 (ΔOR = 0.22). The higher odds of experiencing DD in primary care compared with tertiary care remained significant in the fully adjusted model (OR = 3.68, 95% CI 2.46–5.55; *p* < .001). In addition to care setting, we found four factors independently related to higher DD scores: younger age, participants with dependency on caregivers, higher levels of FBG, and experiencing two or more T2DM complications.

**Table 2:** Results of the ordinal regression models (*n* = 632)

Variables	Models				
	1 pseudo r-square: 0.050	2 pseudo r-square: 0.087	3 pseudo r-square: 0.102	4 pseudo r-square: 0.099	Model 5 pseudo r-square: 0.113
Primary care	2.91 (1.98–4.29)***	3.39 (2.28–5.09)***	3.48 (2.34–5.23)***	3.61 (2.42–5.44)***	3.68 (2.46–5.55)***
Socio-demographic characteristics					
Male sex		1.01 (0.68–1.49)	1.03 (0.69–1.52)	0.98 (0.66–1.45)	1.01 (0.68–1.50)
Age [years]		0.97 (0.96–0.99)***	0.98 (0.96–0.99)**	0.97 (0.96–0.99)***	0.97 (0.96–0.99)**
University degree		1.40 (0.97–2.02)	1.40 (0.97–2.02)	1.38 (0.96–2.00)	1.37 (0.95–1.99)
Occupation (IIvs. I)		1.07 (0.72–1.58)	1.13 (0.76–1.67)	1.08 (0.73–1.60)	1.13 (0.76–1.68)
Occupation (III vs. I)		0.98 (0.62–1.56)	1.01 (0.64–1.61)	0.95 (0.60–1.51)	0.98 (0.62–1.57)
Accompanied by caregiver		1.58 (1.17–2.13)**	1.58 (1.17–2.14)**	1.55 (1.15–2.10)**	1.57 (1.16–2.12)**
Clinical condition					
T2DM duration			0.98 (0.95–1.01)		0.98 (0.96–1.01)
Fasting blood glucose (FBG)			1.01 (1.00–1.01)**		1.01 (1.00–1.01)**
Complications					
With one				1.24 (0.88–1.74)	1.22 (0.87–1.72)
With two or more				1.75 (1.19–2.59)**	1.73 (1.17–2.56)**
With other serious diseases				1.25 (0.67–2.32)	1.11 (0.59–2.08)

Occupation I, II, III respectively stand for active employee, unemployed, and housewife

***P < 0.001;**P < 0.01;*P < 0.05

## Discussion

Our study shows that participants treated in primary care settings indicated more distress on the DDS17 than those who were treated in tertiary care. In addition to the care setting, we found four factors independently related to higher DD scores: younger age, participants with dependency on caregivers, higher levels of FBG, and experiencing two or more T2DM complications. These results need to be interpreted with caution as our data was collected when the Indonesian government initiated a transformation in the health insurance system. Previously, T2DM outpatients were free to choose tertiary services (including choosing a resident of internal medicine). However, the new health insurance system has been further strengthened and referral to health facilities and these changes could very well have an impact on DD.

The association between care setting and DD score was substantially intensified after adjustment for the sociodemographic characteristics (ΔOR = 0.48). This finding is not only attributable to the profound confounding effect of the included factors on the association between the level of healthcare and the DD score, it is also attributable to the highly significant effect of age and dependency on caregivers on the DD scores itself. Specifically, we found that younger age is correlated with higher DD scores even when controlling for the full set of variables (in model 5). This finding is in line with the evidence from several other studies. A comparable study in San Diego stated that the higher DD score in the younger age group may be caused by their family responsibilities, the financial challenges and their daily work [18]. In addition, a study in Malaysia stated that higher DD scores in younger participants were not associated with a higher educational level, but stemmed from the feeling that T2DM disrupted their daily activities due to the therapy and self-management [19]. Furthermore, compared with the elderly, the younger age group has less experience in managing T2DM, specifically, in dealing with the unexpected T2DM diagnosis, therapy and (fear of) complications [20].

We also found that participants with dependency on caregivers had a higher DD score compared to those who were unaccompanied. This finding is obvious to some extent, as the participants dependent on a caregiver were those with poorer health conditions and in need of assistance in activities, such as the elderly or participants with complications or other serious diseases. In addition, most participants with low education stated that they needed a caregiver to assist them during the hospital administration process. In Indonesia, it takes at least 7 h for the patient care process in the hospital starting from registration, laboratory examination and doctor’s consultation until the time they receive their medication from the pharmacist [15]. A caregiver plays a role to help the patients during their treatment in a healthcare facility. Some elderly participants in our study stated that they always forget the physician’s explanation during the consultation after they get back home, but with a caregiver besides them during the consultation, they felt more secure. Furthermore the caregiver could help them to remember the physician’s explanation and could assist in collecting drugs in the pharmacy. Yet, this seems not enough to offset the increased DD scores in this group.

The association between care setting and DD score was slightly intensified after additional adjustment for the factors with regard to the clinical condition (ΔOR = 0.09). This may be partly caused by the limited amount of factors included in this group (i.e., only T2DM duration and FBG level) and the weak but significant effect of the FBG level (OR = 1.01) on DD score. Two more factors regarding clinical condition (type of therapy and postprandial glucose) were initially included in our regression model. However, these two factors were not included in the final models because with these variables included the models no longer met the proportional odds assumption, an important prerequisite to conducting the ordinal regression analysis in a more direct manner [18]. The finding of the association between an elevated FBG level and a higher DD score is in line with other studies. A clinical trial in the U.S. reported that higher levels of blood glucose were associated with higher DD scores. Furthermore, in this U.S. study, it was reported that controlled blood glucose had a positive impact on mood, DD scores and HRQoL [21]. Besides, another study conducted on Hispanic and non-Hispanic patients in U.S also reported that lower DD scores were associated with reductions in blood glucose levels [22]. From an analytical perspective, care has to be taken in this study that due to the high percentage of missing data on the FBG level (258 available evidence out of 632 participants), we used the multiple imputation approach [17] to capture the FBG levels of the total sample. The significant conclusion was then generated based on the total sample instead of the 258 participants who had full evidence of their FBG levels.

The association between care setting and DD score was moderately intensified after additional adjustment for the factors with regard to the complications and other serious diseases (ΔOR = 0.22) within which having two or more complications strongly increased the DD score. This finding is in line with the Dutch study [9] which reported a positive correlation with having different kinds of complications and the increased Problem Areas in Diabetes (PAID) scale. Furthermore, a study in Indian patients also reported that T2DM complications were a major predictor for high DD scores [23]. In our study, 6% of the participants reported other serious diseases (cancer, tuberculosis, gastritis, hepatitis and tumors), resulting in higher DD scores. Research in Greece also reported this positive association between comorbidities and higher DD scores [10].

People living with T2DM require a lifetime daily self-management plan [24]. The changes in their daily lifestyle and the disease may have a negative impact on their psychological state and may contribute to DD. DD refers to the fear of risk of T2DM complications, lack of accessibility to high-quality healthcare facilities, worries about self-management therapy and the perception of lacking emotional support from family and colleagues [5, 7]. Four previous studies in U.S have found that indeed higher DD scores, due to higher levels of distress, were associated with worse psychological outcomes, poor self-care, higher levels of haemoglobin A1c (HbA1c), lower health-related quality of life (HRQoL) and increased T2DM complications [5, 7, 8, 25]. Furthermore, periodic DD assessments are important to facilitate early detection of DD and subsequent potential prevention of more severe psychological disorders; notably, T2DM patients with higher DD scores have been found to have an increased risk of mortality [26]. Regular DD assessment has been recommended by the IDF since 2012 [27].

The higher DD score in primary care found in our study can be explained by several facility-based factors. Recall that our data collection was performed in 2014, during the time when the government of Indonesia began to introduce the new health insurance system transitioning from Askes to BPJS. One of the impacts of this change is that the government strengthened the tiered referral health service process. The health service started from the primary care level, and T2DM outpatients who had been undergoing their therapy at a tertiary care had to be referred back to the primary care (after approval by the internist who takes care of them). In this study, during the data collection process, some of the elderly participants shared their experiences that since the transformation of the health insurance system, they were confused by the complicated procedure and bureaucracy of health care facilities. One of the impacts was a change in the physician that prescribed them the medical treatment. Often, they already felt comfortable with the previous physician who treated them and the change may have caused distress. In addition, some participants argued that T2DM must be treated by specialists (as they had been doing), in other words they were more confident in specialists’ knowledge. Last but not least, some participants also revealed that the laboratory facilities and the medicines at the tertiary care were more complete than the primary care. To some participants, the type of medicine given by the internists was better than the medicine they got from primary care.

Furthermore, as our data collection was conducted in a public area under-reporting of participants’ experience and psychological situation might have occurred due to reluctance of being critical to the authorities. Notably, the above selection criteria on age and willingness to participate were applied. To avoid further selection bias, no other limitations on participation were applied. However, it is important to also note that the specific sites chosen and the type of outpatients that visit the sites did provide a specific selection. Furthermore, during the data collection, the researcher explained to the participants that any answer they provided would be kept private and confidential and an honest or subjective response would be helpful in developing T2DM services in Indonesia.

Limitations of this study were that we were not able to measure the HbA1c of the participants. This is because not all health facilities are equipped with HbA1c testing facilities. For some T2DM outpatients with good economic circumstances, HbA1c testing were conducted in private laboratories. Also, there is a difference in policies on HbA1c tests between different health facilities. As an illustration, one particular PHC recommends only one HbA1c tests per year and on the condition that the T2DM outpatient is participating in activities organized by that particular PHC, whereas Health Minister regulation No.52 of 2016 [28] states that HbA1c tests should be performed every 3 or 6 months. In addition, we also had difficulties in collecting T2DM duration and FBG levels, with only 40–50% of participants having the full evidence. Furthermore, the number of participants in tertiary care was nearly five times higher than in primary care, as primary healthcare facilities seemed reluctant to participate in the study. The unequal sample sizes between groups may influence the accuracy of the T-tests in our descriptive comparisons presented in Table 1. However, as most of the input parameters were not normally distributed and compared using the Kruskal-Wallis test, and it was shown that the Kruskal-Wallis test can be conducted with unequal group sizes [29], we do not think this unequal sample size will influence the statistical power of our model in general. Body mass index was not measured in our study because during the data collection procedure we were told by the participants that they felt uncomfortable about the measurement since they felt too tired because of the bureaucracy in the hospital. Some of them also felt uncomfortable if we measured their weight and height in the hospital waiting room. Another confounding factor, and commonly included in similar studies, is insulin use. We did include this parameter in our descriptive table (Table 1) under ‘type of therapy’. However, type of therapy was not included as a confounder in the ordinal regression model because the inclusion led the model deviating from the proportional odds assumption [16], an assumption that has to be met when using an ordinal regression model in any analysis. Another limitation of this study lies in the collection of clinical samples from our patients. Due to logistic and organizational challenges, not all variables were available for all participants, which limited the possibilities for correcting and adjusting the results. The difference between DD scores in primary care and tertiary care could have been better explained if more confounding factors had been included compared to the current inclusion in our regression models.

It is important to highlight that the majority of the participants involved in this study were those who generally consistently followed the medication treatment, were compliant and regularly visited the health care facilities to get T2DM medicines. We assume this condition is reflected in our research results, that no participants are found with major DD. Further research should be directed to complementarily analyse DD scores in non-compliant and less controlled T2DM patients. In addition to that, we assume if data collection is done in several remote areas in Indonesia, transportation costs will be one of the main problems for them. As an illustration, T2DM outpatients in one of the remote areas (Mansalean village) in Central Sulawesi may spend around USD$10 reaching a health facility.

This is the first study to present DD scores in Indonesia but it is limited to Java. Participants from our study were recruited from various healthcare providers such as family doctors, the T2DM outpatient community, PHCs and hospitals so that we assume that the results of the study may provide an overall understanding of the state of DD in Indonesia. Moreover, although the Indonesian population is very heterogeneous, the Java population can be regarded as quite representative as 57% of all Indonesians reside in Java [30].

It can be implied from this research that serious consideration should be given to conducting T2DM and DD screening within the general context of Indonesian healthcare [31]. Screening is in particular warranted as the management of diabetes is continuously extending and improving, potentially with future consideration of DD being included. The risk of developing T2DM complications can be lowered by population-based prevention programmes (screening regularly for T2DM in people at high risk). Obviously, the cost-effectiveness of the approach should be analyzed. Meanwhile, identified T2DM can be managed through several approaches, such as treatment and/or advice following early detection and diagnosis to prevent rapid complications of T2DM, providing easy access to integrative health facilities including psychological services, essential medicine and basic T2DM technologies [31]. Community support also contributes positively to DD, for example, aiding T2DM patients in accessing healthy food and sports facilities [32, 33], illustrating the need for aligned health literacy programs for the patient and the environment, such as the partners and other family members. We therefore recommend that besides improving access to good health services for those with T2DM, primary care should be comprehensively strengthened in terms of the management of T2DM therapy. Furthermore, we recommend that the Indonesian government should provide psychological help and information in every healthcare facility to help T2DM patients with DD, and inclusive empowerment in the area of health literacy. Such psychological and information services could be embedded within a number of yet small-scale DM club activities (for example Prolanis BPJS). Further (preferably cost-effective) upscaling of these initiatives could be considered.

As one specific initiative, Prolanis concerns a chronic diseases management program organized by the BPJS that facilitates monthly visits between patients and a physician or a consulting resident of internal medicine. In these visits, the patient’s blood glucose is examined, followed by exercise and education about T2DM [34]. In Prolanis activities, doctors could play a role in providing T2DM education and information, while psychologists could provide psychological education to reduce DD. Another specific recommendation is that T2DM education should also be aimed at caregivers or family members because they are the ones who are able to monitor the developments of the therapies given to the T2DM patients, the disease course and the overall well-being. Such increasing awareness and education will empower health literacy of both the patients as well as the environment. It is likely that increasing awareness and educating Indonesians with T2DM not only about DM care but also about the reforms in the health insurance system and healthcare provision may be beneficial in reducing DD. Currently, DD screening has not become a priority in Indonesia even under the recommendation of the IDF [35] and American Diabetes Association [36] as being a global guideline for T2DM. If screening for DM is undertaken, DD should be one of the concerns of the Indonesian government. Additionally, we also recommend gender-specific approaches such as psychological consultations for female T2DM patients, especially housewives. Finally, educating T2DM outpatients about the reforms in the health insurance system and healthcare provision as well as engaging the family members in T2DM education may be beneficial to reduce DD.

## Conclusions

In this study, we found a higher DD score in Indonesian T2DM outpatients from primary care compared to the patients managed in tertiary care. In addition to the care settings, the following variables were found to be positively related to a higher DD score: younger age, participants with dependency on caregivers, higher levels of FBG, and experiencing two or more T2DM complications. This is the first study in Indonesia to compare DD scores within different healthcare facilities. We recommend the general consideration of DD by the government and of various patient characteristics. Our DD-estimates can fruitfully be used in Indonesian healthcare policy making for T2DM patients. Regular DD assessment with good data management can be a reference for the government to determine the intervention type which is suitable for each level of health facility. For example, when there are more T2DM patients with a high DD score in the regimen distress domain in a health care facility, T2DM education and training could be improved. If such actions are taken on the knowledge gathered on DD, important further improvements in diabetes care in Indonesia can be achieved.

## Supplementary information

**Additional file 1.**

**Additional file 2.**

## Data Availability

The datasets used and/or analyzed during the current study are available from the corresponding author on reasonable request.

## Abbreviations

BMI: Body mass index
BPJS: Badan Penyelenggara Jaminan Sosial (social security administrative agency)
DD: Diabetes distress
DDS17 Bahasa Indonesia: Diabetes Distress Scale questionnaire in Bahasa Indonesia
DM: Diabetes Mellitus
FBG: Fasting blood glucose
GP: General practitioner
HbA1c: Haemoglobin A1C
HRQoL: Health-related quality of life (HRQoL)
IDF: International Diabetes Federation
OAD: Oral antidiabetic drug
OR: Odds ratio
PAID: Problem Areas in Diabetes
PHC: Primary healthcare center
T2DM: Type 2 Diabetes Mellitus
ΔOR: The differences in odds ratios between the models
