# Supplementary material for: Diabetes distress in Indonesian patients with type 2 diabetes: a comparison between primary and tertiary care
Source: BMC Health Serv Res. 2019 Oct 30;19:773. doi: 10.1186/s12913-019-4515-1 (PMC6820962; doi:10.1186/s12913-019-4515-1)
Supplement: Supplementary file 1 — Additional file 1. [file 12913_2019_4515_MOESM1_ESM.doc]

**Additional file 1**

**DDS**

**DIRECTIONS:** Living with diabetes can sometimes be tough. There may be many problems and hassles concerning diabetes and they can vary greatly in severity. Problems may range from minor hassles to major life difficulties. Listed below are 17 potential problem areas that people with diabetes may experience. Consider the degree to which each of the 17 items may have distressed or bothered you DURING THE PAST MONTH and circle the appropriate number.

Please note that we are asking you to indicate the degree to which each item may be bothering you in your life, NOT whether the item is merely true for you. If you feel that a particular item is not a bother or a problem for you, you would circle "1". If it is very bothersome to you, you might circle "6".

|  | **Not a Problem** | **A Slight Problem** | **A Moderate Problem** | **Somewhat Serious Problem** | **A Serious Problem** | **A Very Serious Problem** |
| --- | --- | --- | --- | --- | --- | --- |
| 1. Feeling that my doctor doesn't know enough about diabetes and diabetes care. | 1 | 2 | 3 | 4 | 5 | 6 |
| 2. Feeling that diabetes is taking up too much of my mental and physical energy every day. | 1 | 2 | 3 | 4 | 5 | 6 |
| 3. Not feeling confident in my day-to-day ability to manage diabetes. | 1 | 2 | 3 | 4 | 5 | 6 |
| 4. Feeling angry, scared and/or depressed when I think about living with diabetes. | 1 | 2 | 3 | 4 | 5 | 6 |
| 5. Feeling that my doctor doesn't give me clear enough directions on how to manage my diabetes. | 1 | 2 | 3 | 4 | 5 | 6 |
| 6. Feeling that I am not testing my blood sugars frequently enough. | 1 | 2 | 3 | 4 | 5 | 6 |
| 7. Feeling that I will end up with serious long-term complications, no matter what I do. | 1 | 2 | 3 | 4 | 5 | 6 |
| 8. Feeling that I am often failing with my diabetes routine. | 1 | 2 | 3 | 4 | 5 | 6 |

|  | **Not a Problem** | **A Slight Problem** | **A Moderate Problem** | **Somewhat Serious Problem** | **A Serious Problem** | **A Very Serious Problem** |
| --- | --- | --- | --- | --- | --- | --- |
| 9. Feeling that friends or family  are not supportive enough of  self-care efforts (e.g. planning activities that conflict with my  schedule, encouraging me to  eat the "wrong" foods). | 1 | 2 | 3 | 4 | 5 | 6 |
| 10. Feeling that diabetes controls my life. | 1 | 2 | 3 | 4 | 5 | 6 |
| 11. Feeling that my doctor doesn't take my concerns seriously enough. | 1 | 2 | 3 | 4 | 5 | 6 |
| 12. Feeling that I am not sticking closely enough to a good meal plan. | 1 | 2 | 3 | 4 | 5 | 6 |
| 13. Feeling that friends or family don't appreciate how difficult living with diabetes can be. | 1 | 2 | 3 | 4 | 5 | 6 |
| 14. Feeling overwhelmed by the demands of living with diabetes. | 1 | 2 | 3 | 4 | 5 | 6 |
| 15. Feeling that I don't have a doctor who I can see regularly enough about my diabetes. | 1 | 2 | 3 | 4 | 5 | 6 |
| 16. Not feeling motivated to keep up my diabetes self­ management. | 1 | 2 | 3 | 4 | 5 | 6 |
| 17. Feeling that friends or family don't give me the emotional support that I would like. | 1 | 2 | 3 | 4 | 5 | 6 |

**DDS17 SCORING SHEET**

INSTRUCTIONS FOR SCORING:

The DDS17 yields a total diabetes distress score plus 4 subscale scores, each addressing a different kind of distress.^1^ To score, simply sum the patient’s responses to the appropriate items and divide by the number of items in that scale.

Current research^2^ suggests that a mean item score 2.0 – 2.9 should be considered ‘moderate distress,’ and a mean item score > 3.0 should be considered ‘high distress.’ Current research also indicates that associations between DDS scores and behavioral management and biological variables (e.g., A1C) occur with DDS scores of > 2.0. Clinicians may consider moderate or high distress worthy of clinical attention, depending on the clinical context.

We also suggest reviewing the patient’s responses across all items, regardless of mean item scores. It may be helpful to inquire further or to begin a conversation about any single item scored > 3.

Total DDS Score: a. Sum of 17 item scores. ______________

b. Divide by: _____17_______

c. Mean item score: ______________

Moderate distress or greater? (mean item score > 2) yes__ no__

A. Emotional Burden: a. Sum of 5 items (2, 4, 7, 10, 14) _______________

b. Divide by: _______5_______

c. Mean item score: ______________

Moderate distress or greater? (mean item score > 2) yes__ no__

B. Physician Distress: a. Sum of 4 items (1, 5, 11, 15) ______________

b. Divide by: _______4______

c. Mean item score: ______________

Moderate distress or greater? (mean item score > 2) yes__ no__

C. Regimen Distress: a. Sum of 5 items (6, 8, 3, 12, 16) ______________

b. Divide by: _______5______

c. Mean item score: ______________

Moderate distress or greater? (mean item score > 2) yes__ no__

D. Interpersonal Distress: a. Sum of 3 items (9, 13, 17) ­­­­­­______________

b. Divide by: _______3______

1. Mean item score: ______________

Moderate distress or greater? (mean item score > 2) yes__ no__

1. Polonsky, W.H., Fisher, L., Esarles, J., Dudl, R.J., Lees, J., Mullan, J.T., Jackson, R. (2005). Assessing psychosocial distress in diabetes: Development of the Diabetes Distress Scale. Diabetes Care, 28, 626-631.

2. Fisher, L., Hessler, D.M., Polonsky, W.H., Mullan, J. (2012). When is diabetes distress clinically meaningful? Establishing cut-points for the Diabetes Distress Scale. Diabetes Care, 35, 259-264.
