# Supplementary material for: Diabetes distress in Indonesian patients with type 2 diabetes: a comparison between primary and tertiary care
Source: BMC Health Serv Res. 2019 Oct 30;19:773. doi: 10.1186/s12913-019-4515-1 (PMC6820962; doi:10.1186/s12913-019-4515-1)
Supplement: Supplementary file 2 — Additional file 2. [file 12913_2019_4515_MOESM2_ESM.docx]

**Additional file 2**

**DDS**

PETUNJUK : Hidup dengan menderita **penyakit diabetes (sakit gula atau kencing manis)** terkadang sulit. Banyak permasalahan dan gangguan terkait dengan diabetes, dan penyakit tersebut bisa bervariasi tergantung pada tingkat keparahannya. Permasalahan mungkin datang dari gangguan kecil hingga kesulitan besar dalam hidup.

Berikut ini adalah 17 (tujuh belas) masalah yang mungkin dialami penderita diabetes.

Pertimbangkan seberapa jauh ke 17 masalah tersebut telah mengganggu dan menekan hidup anda selama **1 (SATU) BULAN TERAKHIR**.

Harap diingat bahwa kami meminta Anda untuk menunjukkan sejauh mana masing-masing tersebut mengganggu kehidupan anda, BUKAN apakah pernyataan tersebut semata-mata benar untuk Anda.

Jika Anda merasa bahwa suatu pernyataan pada tabel di bawah ini,

**Bukanlah suatu gangguan atau masalah, Anda bisa melingkari angka “1”.**

**Namun, jika hal tersebut sangatlah mengganggu, Anda dapat melingkari angka “6”.**

| No |  | Bukan Masalah | Masalah Ringan | Masalah Sedang | Masalah Cukup Serius | Masalah Serius | Masalah Sangat Serius |
| --- | --- | --- | --- | --- | --- | --- | --- |
| 1. | Saya merasa bahwa teman-teman atau keluarga saya tidak memberikan dukungan emosional yang saya inginkan.  Contoh dukungan emosional: misalnya mereka selalu mengingatkan saya, agar makan makanan yang baik, olah raga, mengingatkan minum obat dan menjaga kebersihan. | 1 | 2 | 3 | 4 | 5 | 6 |
| 2. | Saya merasa bahwa teman-teman atau keluarga tidak menghargai bagaimana sulitnya hidup dengan diabetes. | 1 | 2 | 3 | 4 | 5 | 6 |
| 3. | Saya merasa bahwa teman-teman atau keluarga saya tidak cukup mendukung usaha perawatan mandiri (contohnya: mereka tetap mengajak saya makan makanan yang salah, misalnya mengajak makan direstoran dengan makanan berlemak atau yang manis-manis). | 1 | 2 | 3 | 4 | 5 | 6 |
| 4. | Saya merasa tidak mempunyai dokter yang bisa saya temui secara teratur untuk berkonsultasi masalah diabetes. | 1 | 2 | 3 | 4 | 5 | 6 |
| 5. | Saya sendiri merasa tidak termotivasi untuk meneruskan penanganan diabetes. | 1 | 2 | 3 | 4 | 5 | 6 |
| 6. | Saya merasa marah, takut dan/atau tertekan ketika saya memikirkan tentang hidup dengan menderita diabetes. | 1 | 2 | 3 | 4 | 5 | 6 |
|  |  | Bukan Masalah | Masalah Ringan | Masalah Sedang | Masalah Cukup Serius | Masalah Serius | Masalah Sangat Serius |
| 7. | Saya merasa diabetes mengambil terlalu banyak energi jiwa dan fisik setiap harinya. | 1 | 2 | 3 | 4 | 5 | 6 |
| 8. | Saya merasa kewalahan oleh tuntutan hidup dengan penyakit diabetes | 1 | 2 | 3 | 4 | 5 | 6 |
| 9. | Saya merasa bahwa nantinya dalam hidup saya, saya akan mengalami komplikasi serius jangka panjang, terlepas dari apapun yang saya lakukan. | 1 | 2 | 3 | 4 | 5 | 6 |
| 10. | Saya merasa tidak percaya diri dengan kemampuan keseharian saya dalam menangani masalah diabetes.  Contohnya: menjaga pola makan dan kebersihan, minum obat tepat waktu dan olah raga teratur. | 1 | 2 | 3 | 4 | 5 | 6 |
| 11. | Saya merasa bahwa dokter saya tidak cukup mengetahui tentang perawatan diabetes. | 1 | 2 | 3 | 4 | 5 | 6 |
| 12. | Saya merasa bahwa dokter tidak memberikan petunjuk yang cukup jelas tentang bagaimana menangani diabetes. | 1 | 2 | 3 | 4 | 5 | 6 |
| 13. | Saya merasa dokter tidak cukup serius dalam memperhatikan kekhawatiran  Yang saya rasakan. | 1 | 2 | 3 | 4 | 5 | 6 |
| 14. | Saya merasa bahwa saya tidak cukup sering melakukan pengetesan gula darah. | 1 | 2 | 3 | 4 | 5 | 6 |
| 15. | Saya merasa bahwa saya sering gagal dengan rutinitas diabetes saya, misalnya saya sering lupa minum obat atau merasa malas mengecek gula darah. | 1 | 2 | 3 | 4 | 5 | 6 |
| 16. | Saya merasa bahwa saya tidak ketat dalam menyiapkan makanan yang baik | 1 | 2 | 3 | 4 | 5 | 6 |
| 17. | Saya merasa bahwa diabetes mengontrol hidup saya, dimana saya merasa bahwa aktivitas aktivitas saya menjadi terbatas sejak dan selama saya menderita diabetes. | 1 | 2 | 3 | 4 | 5 | 6 |

LEMBAR PENILAIAN DDS17

PETUNJUK PENILAIAN:

DDS17 menggambarkan total gangguan yang dialami penderita diabetes ditambah 4 domain dimana tiap domain tersebut menunjukkan jenis gangguan yang berbeda.

Penilaian dilakukan dengan cara sederhana yaitu dengan menjumlahkan jawaban-jawaban pasien pada pernyataan yang sesuai dan membagi dengan jumlah pernyataan yang ada pada domain itu.

Sejumlah riset terbaru menyebutkan bahwa jika nilai tengah dari hasil perhitungan adalah:

**2.0 sampai 2.9 : kesulitan yang dialami penderita diabetes berada pada tingkat sedang**

**≥3.0 : kesulitan yang dialami penderita diabetes berada pada tingkat tinggi**

Penelitian terbaru lainnya, mengindikasikan bahwa hubungan antara hasil penilaian DDS17 Bahasa Indonesia, manajemen perilaku dan variabel biologis (misalnya: hasil pemeriksaan HbA1c) terjadi pada nilai DDS ≥2.0. Para klinisi dapat memberikan perhatian klinis yang lebih pada penderita diabetes dengan tingkat kesulitan sedang hingga tinggi, tergantung dari kondisi klinis pendeita.

Kami juga menyarankan untuk meninjau kembali jawaban pasien atas semua pernyataan, tanpa melihat nilai rata-ratanya. Hal ini akan sangat membantu dalam mengidentifikasi masalah lebih tajam atau memulai wawancara tentang setiap pernyataan dengan nilai ≥3.

Nilai Total DDS :

Total jumlah nilai dari 17 pernyataan _____________

Dibagi dengan: ______17_____

Nilai rata-rata pernyataan: _____________

Kesulitan sedang atau lebih besar? (nilai rata-rata >2) Ya___Tidak___

**A. Beban Emosional**

a. Jumlah dari 5 pernyataan (6, 7, 8, 9, 17) _____________

b. Dibagi dengan: ______5_____

c. Nilai rata-rata pernyataan: _____________

Kesulitan Sedang atau lebih besar? (nilai rata-rata >2) Ya___Tidak___

**B. Kesulitan dengan dokter**

a. Jumlah dari 4 pernyataan (4, 11, 12, 13) _____________

b. Dibagi dengan ______4______

c. Nilai rata-rata pernyataan: _____________

Kesulitan Sedang atau lebih besar? (nilai rata-rata >2) Ya___Tidak___

**C. Kesulitan dalam manajemen terapi diabetes**

a. Jumlah dari 5 pernyataan (5, 10, 14, 15, 16,) _____________

b. Dibagi dengan ______5______

c. Nilai rata-rata pernyataan: _____________

Kesulitan Sedang atau lebih besar? (nilai rata-rata >2) Ya___Tidak___

**D. Kesulitan dengan Keluarga, teman dan orang-orang sekitar**

a. Jumlah dari 3 pernyataan (1, 2, 3) _____________

b. Dibagi dengan ______3______

c. Nilai rata-rata pernyataan: _____________

Kesulitan Sedang atau lebih besar? (nilai rata-rata >2) Ya___Tidak___

Sumber Pustaka

1. Polonsky, W.H., Fisher, L., Esarles, J., Dudl, R.J., Lees, J., Mullan, J.T., Jackson, R. (2005). Assessing psychosocial distress in diabetes: Development of the Diabetes Distress Scale. Diabetes Care, 28, 626-631.

2. Fisher, L., Hessler, D.M., Polonsky, W.H., Mullan, J. (2012). When is diabetes distress clinically meaningful? Establishing cut-points for the Diabetes Distress Scale. Diabetes Care, 35, 259-264.

3. Arifin B, Perwitasari DA, Cao Q, Atthobari J, Krabbe PF., Postma MJ. Translation, revision and validation of the diabetes distress scale for Indonesian type 2 diabetic outpatients with various types of complications. Value Heal Reg Issues. 2017;12C:63–73.
